# Risk of Major Congenital Malformations Associated With the First‐Trimester Exposure to Antipsychotics: A Large Claims Database Study

**DOI:** 10.1002/prp2.70231

**Published:** 2026-03-11

**Authors:** Ryo Obara, Takamasa Sakai, Tomofumi Ishikawa, Kei Morishita, Motohiko Adomi, Azusa Hara, Saya Kikuchi, Natsuko Kobayashi, Noriyuki Iwama, Genki Shinoda, Aoi Noda, Masatsugu Orui, Mami Ishikuro, Hiroaki Tomita, Nariyasu Mano, Shinichi Kuriyama, Taku Obara

**Affiliations:** ^1^ Division of Molecular Epidemiology, Graduate School of Medicine Tohoku University Sendai Miyagi Japan; ^2^ Drug Informatics, Faculty of Pharmacy Meijo University Nagoya Aichi Japan; ^3^ Laboratory of Biomolecule and Pathophysiological Chemistry, Graduate School of Pharmaceutical Sciences Tohoku University Sendai Miyagi Japan; ^4^ Department of Pharmaceutical Sciences Tohoku University Hospital Sendai Miyagi Japan; ^5^ Department of Epidemiology Harvard T.H. Chan School of Public Health Boston Massachusetts USA; ^6^ Laboratory of Social Pharmacy and Epidemiology Showa Pharmaceutical University Machida Tokyo Japan; ^7^ Department of Psychiatry Tohoku University Hospital Sendai Miyagi Japan; ^8^ Department of Psychiatry, Graduate School of Medicine Tohoku University Sendai Miyagi Japan; ^9^ Department of Preventive Medicine and Epidemiology, Tohoku Medical Megabank Organization Tohoku University Sendai Miyagi Japan; ^10^ Center for Maternal and Perinatal Medicine Tohoku University Hospital Sendai Miyagi Japan; ^11^ Department of Disaster Public Health, International Research Institute of Disaster Science Tohoku University Sendai Miyagi Japan; ^12^ Department of Disaster Psychiatry, International Research Institute of Disaster Science Tohoku University Sendai Miyagi Japan

**Keywords:** antipsychotics, claims database, congenital malformations, first‐trimester exposure, pharmacoepidemiology, pregnancy

## Abstract

This study aimed to evaluate the risk of major congenital malformations (MCMs) associated with first‐trimester exposure to antipsychotics, using a large claims database in Japan. This retrospective cohort study was based on data from a large claims database in Japan. We used a large claims database from 2010 to 2019. Dates of pregnancy onset and delivery were estimated using the developed algorithms. Overall MCMs were defined according to the International Classification of Diseases, 10th revision codes. To address confounding by indication, the risk of MCM in relation to first‐trimester antipsychotic use was evaluated among pregnant women with psychiatric disorders diagnosed before the end of the first‐trimester. We compared the prevalence of MCMs among infants born to pregnant women with or without first‐trimester exposure to antipsychotics and estimated the weighted odds ratios (wORs) using propensity‐score overlap weights. The prevalence of psychiatric diagnoses before the end of the first‐trimester was 6291 women among the 91 390 who delivered between 2010 and 2019. Among the 6291 diagnosed with psychiatric disorders, 317 were exposed to any antipsychotics during the first‐trimester of pregnancy. The prevalence of overall MCMs among live births was 411 (6.9%) among women unexposed to any antipsychotics and 24 (7.6%) among those exposed. The first‐trimester exposure to any antipsychotics was not significantly associated with overall MCMs when wORs were calculated using propensity‐score overlap weights (wOR 1.144, 95% confidence intervals 0.727–1.799). Exposure to any antipsychotics during the first‐trimester of pregnancy was not associated with an increased risk of overall MCMs in infants.

**Abbreviations**
ACOG: American College of Obstetricians and Gynecologists
CIs: confidence intervals
ICD‐10: International Classification of Diseases, 10th revision
MCMs: major congenital malformations
ORs: odds ratios
SMD: standardized mean difference
wORs: weighted odds ratios

## 1 Introduction

Psychiatric disorders are common in women of reproductive age and occur frequently during pregnancy [1, 2, 3]. Pregnant women with psychiatric disorders are at risk of worsening symptoms and perinatal complications due to untreated or insufficiently treated conditions [4, 5]. According to the American College of Obstetricians and Gynecologists (ACOG) Clinical Practice Guideline No. 4, untreated and undertreated mental health conditions can have significant negative effects that can be mitigated with timely treatment, especially when initiated early [6]. The ACOG Guideline No. 5 recommends optimal dosing to prevent understatement when pharmacotherapy is initiated or continued, following discussions about psychotherapy and adjunctive care [7]. According to the Perinatal Mental Health Consensus Guide 2023 in Japan [8], patients undergoing psychiatric drug therapy should continue taking the drug during pregnancy because symptoms may recur if the drug is discontinued. However, contrary to these recommendations, psychiatric medication prescriptions are usually discontinued during pregnancy [9, 10, 11], though it is unclear whether this trend necessarily follows current clinical guidelines. Moreover, one of the reasons is the influence of drug package inserts. Some antipsychotics, such as haloperidol and bromperidol, are contraindicated in pregnant women due to their teratogenic effect in animal experiments [12, 13]. This information may raise concerns about overall antipsychotic use during pregnancy. Another reason for the discontinuation of treatment during pregnancy is insufficient evidence on the effects of psychiatric medication use during pregnancy on mothers and children, which may lead to anxiety among both physicians and mothers about whether to prescribe and take psychiatric medications. In Japan, information regarding the safety of psychiatric medications, particularly antipsychotics, during pregnancy is limited. For example, although studies in Japan have examined antidepressant use and major congenital malformations (MCMs) [14, 15], to the best of our knowledge, only one study has investigated the use of MCMs to the best of our knowledge [16]. No research on antipsychotics cited in the Japanese guide comes from Japan. Ethnic differences between the Japanese and non‐Japanese populations may influence drug metabolism [17]. The availability of antipsychotics and prescription trends vary across countries [18, 19, 20, 21]. Given the differences in ethnicity and medical care delivery environments, using data corresponding to Japanese real‐world settings is necessary [22].

While evidence on antipsychotics' teratogenic risk is accumulating in Europe and the United States [18], there is only one study conducted in Japan at the National Center for Child Health and Development using the Teratology Information Service database with a healthy comparison group of women unexposed to teratogenic medication [16]. To address confounding by indication in such study design [16], it is more meaningful to use a comparison group of women with histories of psychiatric illness who are not taking atypical antipsychotics rather than using a healthy comparison group of women unexposed to teratogenic medication [23]. Previous studies have shown the potential impact of maternal psychiatric disorders on maternal and infant outcomes [24, 25, 26], necessitating the consideration of psychiatric disorders to clarify the association between antipsychotic exposure during pregnancy and infant outcomes.

Our study used a large claims database in Japan to evaluate the risk of MCMs associated with antipsychotic exposure during the first trimester among patients with psychiatric disorders. We hypothesized that there would be no significant association between antipsychotic use during the first trimester of pregnancy and the risk of MCMs in newborns.

## 2 Methods

### 2.1 Data Source

A large administrative claims database from JMDC Inc. (Tokyo, Japan) [27], containing all inpatient, outpatient, and pharmacy claims received from insurers, was used. These claims include diagnoses, surgical and medical procedures, and dispensed medication. Consistent health insurance ensures data recording, irrespective of patient transfers between hospitals or facilities. Standardized disease classifications and anonymous record linkages were used in the database [27]. Details on the database are described in previous reports [21, 27, 28, 29, 30, 31, 32, 33].

### 2.2 Study Population

**FIGURE 1:**
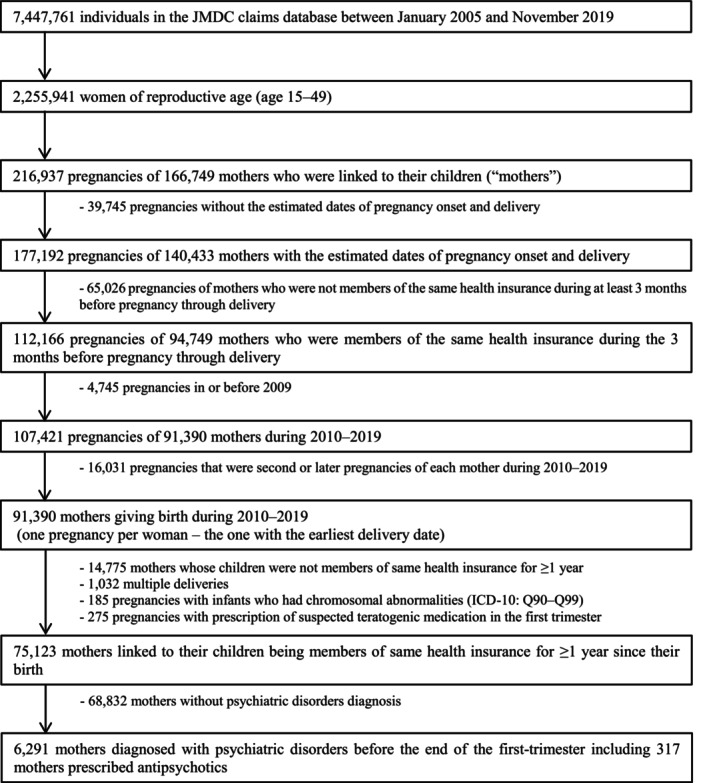
Flow chart of the selection of the study population for data analysis.

The dataset available on May 8, 2020, included data on 7 447 761 individuals covered by health insurers between January 2005 and November 2019 (Figure 1). To evaluate the effects of drugs on congenital malformations, we identified mother‐infant pairs using the following algorithm [30, 31, 32, 33]. Mothers are linked to offspring if they are enrolled with the same health insurer, allowing identification of the birth month and year. Women who met the following eligibility criteria were selected as the study population to evaluate antipsychotic exposures during pregnancy: those whose infants were enrolled with the same health insurer, pregnancy onset and delivery dates were estimable, and who were enrolled with the health insurers continuously from 3 months before the onset of pregnancy until delivery. Due to the limited number of women who gave birth before 2009, we included only the first pregnancy of each woman between 2010 and 2019. We selected mothers whose infants were enrolled with the same health insurer for at least 1 year from their birth month. We excluded mothers with multiple deliveries, as multiple births are associated with an increased risk of congenital disabilities [34], and excluded infants with chromosomal abnormalities (International Classification of Diseases, 10th revision [ICD‐10] code Q90–Q99) since these are not because of exposure. Including them would reduce the likelihood of detecting exposure‐related risk [35]. We also excluded pregnancies if the mother was prescribed any known teratogenic medication during the first trimester (etretinate, carbamazepine, thalidomide, cyclophosphamide, danazol, thiamazole, trimethadione, valproate, vitamin A [retinol], phenytoin, phenobarbital, mycophenolate, misoprostol, methotrexate, warfarin) [36].

To address confounding by indication, the study population was restricted to women diagnosed with psychiatric disorders. In this study, psychiatric disorders were defined as mental and behavioral disorders (ICD‐10 F00–99) diagnosed before the end of the first trimester, including the pre‐pregnancy period. This category includes major psychiatric conditions for which antipsychotics are indicated, such as schizophrenia (F20), bipolar disorder (F31), and depressive episodes (F32).

### 2.3 Estimation of Pregnancy Onset and Delivery

No dates of pregnancy onset or delivery were available in the Japanese claims database. Thus, the dates were estimated using algorithms described elsewhere [11, 28, 29], the protocol detailed in Data S1 [36, 37, 38].

### 2.4 Antipsychotic Exposure

In this study, antipsychotics were identified by their nonproprietary names, and those evaluated are listed in Table S1, which includes all antipsychotics approved in Japan at the time of prescribing. Among these, exposure to any antipsychotics was assessed during the first trimester; in addition, exposures to any typical antipsychotics and any atypical antipsychotics were evaluated separately. Exposure to individual antipsychotics was also evaluated separately. The dispensing date was preferentially used to estimate the timing of exposure; however, if unavailable, the hospitalization date was used instead because it was the only other specific date available for drug data. With the month and year available for each claim, drugs were assigned to the 15th of the month if the dispensing or hospitalization date was unavailable. The days of supply for each prescription were considered to determine the timing of exposure, as women might still be exposed within several days from the prescription. Antipsychotics were classified according to their route of administration and generic names.

### 2.5 MCM Outcomes

The diagnosis of overall MCMs in the first year of life was identified according to ICD‐10 codes [32, 39, 40, 41], namely, ICD‐10 codes Q00–Q89, excluding minor congenital malformations (Q10, Q162, Q17–Q182, Q184–Q189, Q250, Q270, Q381, Q515, Q516, Q520–Q527, Q53, Q664–Q666, Q69, Q70, Q81–Q84). The MCMs in claims were validated against patient medical records in a university hospital, and the overall positive predictive value of the MCMs was 91.5% [39].

### 2.6 Covariates

The covariates considered in the adjustment for confounding variables are listed in Table S2. They were considered a cause of the outcome or both exposure and outcome [42]. The definitions of the covariates were similar to those used in previous studies [21, 30, 31, 32, 33, 35, 36, 43, 44, 45, 46, 47].

To evaluate the risk of MCMs in infants between pregnant women with and without antipsychotic exposure, we used 21 groups of diseases from ICD‐10 chapters as covariates, excluding F00‐F99 (mental and behavioral disorders). These covariates were selected to cover various diseases and conditions that could affect maternal health and pregnancy outcomes, excluding overall MCMs in infants. This approach allows for appropriate adjustment of covariate effects, enabling accurate and comprehensive risk assessment.

### 2.7 Data Analyses

Baseline characteristics were compared between groups with and without antipsychotic exposure during the first‐trimester. The standardized mean differences (SMD) between groups were calculated, and an SMD greater than 10% was considered an indicator of imbalance between groups.

To adjust for confounding factors at baseline, we used propensity‐score overlap weights [48, 49]. First, we calculated the propensity scores for exposure to antipsychotics during the first trimester. Propensity scores were predicted using all covariates listed in Table S2 using a logistic regression model. Second, overlap weights were calculated as the probability that the group was assigned to the opposite group. Overlap weights downweigh individuals in the tails of the propensity‐score distribution and create a balance between the treatment groups regarding all covariates included in the propensity‐score models [48].

Logistic regression analysis was used to estimate the odds ratios (ORs) for overall MCMs and their corresponding 95% confidence intervals (CIs), comparing those with both a diagnosis and antipsychotic exposure to those with a diagnosis but without exposure. The results are provided for the unweighted and overlap‐weighted analyses. To account for weighting, robust variance was used to estimate the 95% CIs. When performing logistic regression analysis, we also applied the 10‐events‐per‐variable rule by Peduzzi et al. [50]. To ensure sufficient stability of model estimates. We limited the analysis to cases in which either the exposed or unexposed group had at least 10 outcome events.

To check the robustness of the results, we conducted several sensitivity analyses for the overall MCMs in the study populations (details are provided in the Data S1). Differences were considered significant when the 95% CIs did not overlap by 1.0. All data were analyzed using SAS version 9.4 (SAS Institute Inc., NC, USA).

## 3 Results

### 3.1 Risk of Overall MCMs Associated With the First‐Trimester Exposure to Any Antipsychotics With Psychiatric Disorders

The study population comprised 91 390 women (Figure 1), and their delivery dates were estimated using the methods described in a database study [30, 31, 32, 33]. Among the 75 398 women, 6291 were diagnosed with psychiatric disorders before the end of the first‐trimester, and 317 were exposed to antipsychotics during the first‐trimester (Figure 1, Table 1). In this section, we analyze the association between first‐trimester exposure to antipsychotics and the risk of overall MCM in pregnant women diagnosed with psychiatric disorders. Pregnant women exposed to antipsychotics were more likely to have diabetes, obesity, epilepsy, a higher count of distinct diagnoses before the end of the first‐trimester, and a greater number of distinct prescribed medications during the first‐trimester than those without antipsychotic exposure (Table 1). After applying overlap weights, all covariates included in the propensity‐score models were balanced (all post‐weighting SMDs < ±0.0001, i.e., substantially below the ±0.1 threshold for imbalance). Psychiatric disorders requiring antipsychotic treatment were more prevalent in the exposed group. Specifically, 70.7% of women exposed to antipsychotics had schizophrenia (F20), 25.2% had bipolar disorder (F31), and 53.6% experienced depressive episodes (F32), compared with 5.0%, 4.4%, and 30.2%, respectively, in the unexposed group (Table S3). Regarding exposure prevalence in the first trimester, the most common atypical antipsychotic monotherapy was oral aripiprazole, followed by oral olanzapine, quetiapine, risperidone, and blonanserin (Table S4). The most common typical antipsychotic monotherapy is oral sulpiride, followed by oral chlorpromazine, levomepromazine, haloperidol, and bromperidol. Haloperidol or bromperidol, both of which are of concern because of their potential teratogenicity, are contraindicated for pregnant women in Japan during the first trimester.

**TABLE 1:** Characteristics of women with psychiatric disorders, with or without antipsychotic exposure.

	Women with psychiatric disorders before the end of the first‐trimester								
	Unweighted population					Weighted population^a^			
	Without antipsychotics in the first‐trimester		With antipsychotics in the first‐trimester		SMD^b^	Without antipsychotics in the first‐trimester		With antipsychotics in the first‐trimester	
	(*N* = 5974)		(*N* = 317)		%	(*N* = 272.7)		(*N* = 272.7)	
	*n* (%)		*n* (%)			*n* (%)		*n* (%)	
Maternal age at delivery (years), mean (SD)	32.9	(4.6)	33.1	(4.8)	1.9	33.0	(1.0)	33.0	(4.4)
≦ 24	185	(3.1)	8	(2.5)		9.5	(3.5)	6.8	(2.5)
25–29	1268	(21.2)	76	(24.0)		52.8	(19.4)	65.6	(24.1)
30–34	2266	(37.9)	101	(31.9)		105.3	(38.6)	87.6	(32.1)
≧ 35	2255	(37.7)	132	(41.6)		105.1	(38.6)	112.6	(41.3)
Delivery year					3.7				
2010–2012	792	(13.3)	39	(12.3)		34.3	(12.6)	33.1	(12.1)
2013–2015	2141	(35.8)	102	(32.2)		93.9	(34.4)	87.3	(32.0)
2016–2018	3041	(50.9)	176	(55.5)		144.5	(53.0)	152.2	(55.8)
Diabetes	363	(6.1)	25	(7.9)	7.1	20.8	(7.6)	20.8	(7.6)
Obesity	82	(1.4)	15	(4.7)	19.6	10.1	(3.7)	10.1	(3.7)
Hypertension	186	(3.1)	11	(3.5)	2.0	8.6	(3.1)	8.6	(3.1)
Epilepsy	183	(3.1)	50	(15.8)	44.5	35.3	(13.0)	35.3	(13.0)
Phenylketonuria	0	(0.0)	0	(0.0)	—	0.0	(0.0)	0.0	(0.0)
ICD‐10 chapters									
A00‐B99 Certain infectious and parasitic diseases	4140	(69.3)	200	(63.0)	−13.1	171.9	(63.0)	171.9	(63.0)
C00‐D48 Neoplasms	2076	(34.8)	89	(28.2)	−14.4	78.0	(28.6)	78.0	(28.6)
D50‐D89 Diseases of the blood and blood‐forming organs	2133	(35.7)	77	(24.0)	−25.1	67.5	(24.7)	67.5	(24.7)
E00‐E90 Endocrine, nutritional, and metabolic diseases	3891	(65.1)	222	(69.2)	10.5	187.3	(68.7)	187.3	(68.7)
G00‐G99 Diseases of the nervous system	2728	(45.7)	246	(78.5)	69.5	204.5	(75.0)	204.5	(75.0)
H00‐H59 Diseases of the eye and adnexa	3749	(62.8)	167	(52.0)	−20.5	147.6	(54.1)	147.6	(54.1)
H60‐H95 Diseases of the ear and mastoid process	1655	(27.7)	69	(22.0)	−13.8	61.4	(22.5)	61.4	(22.5)
I00‐I99 Diseases of the circulatory system	941	(15.8)	40	(12.1)	−9.0	35.3	(13.0)	35.3	(12.9)
J00‐J99 Diseases of the respiratory system	5124	(85.8)	242	(75.7)	−24.2	210.9	(77.3)	210.8	(77.3)
K00‐K93 Diseases of the digestive system	4815	(80.6)	259	(82.2)	2.8	221.3	(81.2)	221.3	(81.2)
L00‐L99 Diseases of the skin and subcutaneous tissue	3960	(66.3)	194	(59.9)	−10.6	168.7	(61.9)	168.7	(61.9)
M00‐M99 Diseases of the musculoskeletal system and connective tissue	3151	(52.7)	155	(48.9)	−7.7	133.1	(48.8)	133.1	(48.8)
N00‐N99 Diseases of the genitourinary system	4944	(82.8)	259	(80.5)	−2.8	221.7	(81.3)	221.7	(81.3)
O00‐O99 Pregnancy, childbirth, and the puerperium	4763	(79.7)	252	(78.5)	−0.6	215.2	(78.9)	215.2	(78.9)
P00‐P96 Certain conditions originating in the perinatal period	189	(3.2)	5	(1.7)	−10.4	3.9	(1.4)	3.9	(1.4)
Q00‐Q99 Congenital malformations, deformations, and chromosomal abnormalities	216	(3.6)	8	(2.8)	−6.3	7.1	(2.6)	8.7	(2.6)
R00‐R99 Symptoms, signs, and abnormal clinical and laboratory findings, not elsewhere classified	4216	(70.6)	199	(61.6)	−16.6	172.4	(63.2)	186.5	(63.2)
S00‐T98 Injury, poisoning, and certain other consequences of external causes	1919	(32.1)	101	(31.9)	−0.6	86.7	(31.8)	93.8	(31.8)
U00‐U99 Codes for special purposes	0	(0.0)	0	(0.0)	—	0.0	(0.0)	0.0	(0.0)
V01‐Y98 External causes of morbidity and mortality	0	(0.0)	0	(0.0)	—	0.0	(0.0)	0.0	(0.0)
Z00‐Z99 Factors influencing health status and contact with health services	552	(9.2)	38	(12.0)	8.9	29.9	(11.0)	29.9	(11.0)
No. of diagnoses before the end of the first‐trimester, mean (SD)	18.0	(8.2)	17.7	(8.3)	−3.7	17.6	(2.0)	17.6	(7.7)
No. of prescribed medications in the first‐trimester, mean (SD)	5.7	(5.5)	9.7	(6.9)	63.8	9.1	(1.8)	9.1	(5.9)

Abbreviations: ICD‐10, International Classification of Diseases 10th Revision; SD, Standard deviation; SMD, Standardized mean difference.

^a^ Overlap weights create a perfect balance of mean values of covariates included in the propensity score; therefore, the exposure groups are identical after weighting.

^b^ A standardized difference of less than 10% was considered to be balanced.

The prevalence of overall MCMs among infants born to 6291 mothers with psychiatric disorders was 7.6% (*n* = 24) for 317 mothers exposed to any antipsychotics in the first trimester and 6.9% (*n* = 411) for 5974 mothers unexposed (Table 2).

**TABLE 2:** Prevalence of overall major congenital malformations in the study population with antipsychotic exposure (monotherapy or combination therapy) and without antipsychotic exposure.

Women with psychiatric disorders diagnosed before the end of the first‐trimester	Total no.	No. of events	(%)
Unexposed any antipsychotics	5974	411	(6.9)
Exposed any antipsychotics	317	24	(7.6)
Any typical antipsychotics	66	3	(4.5)
Any typical antipsychotics only^a^	46	2	(4.3)
Any atypical antipsychotics	271	22	(8.1)
Any atypical antipsychotics only^b^	251	21	(8.4)
Oral any antipsychotics	315	24	(7.6)
Oral any typical antipsychotics	65	3	(4.6)
Oral sulpiride	25	0	(0.0)
Oral levomepromazine	16	1	(6.3)
Oral chlorpromazine	12	0	(0.0)
Oral haroperidol	7	1	(14.3)
Oral bromperidol	3	0	(0.0)
Oral prochlorperazine	3	1	(33.3)
Oral fluphenazine	2	0	(0.0)
Oral perphenazine	2	0	(0.0)
Oral propericiazine	1	0	(0.0)
Oral tiapride	1	0	(0.0)
Oral any atypical antipsychotics	270	22	(8.2)
Oral aripiprazole	123	8	(6.5)
Oral quetiapine	59	6	(10.2)
Oral olanzapine	57	3	(5.3)
Oral risperidone	52	5	(9.6)
Oral blonanserin	23	2	(8.7)
Oral perospirone	17	3	(17.7)
Oral paliperidone	3	0	(0.0)
Oral asenapine	1	0	(0.0)
Injectionable any antipsychotics	5	0	(0.0)
Injectionable any typical antipsychotics	3	0	(0.0)
Injectionable haroperidol	3	0	(0.0)
Injectionable any atypical antipsychotics	3	0	(0.0)
Injectionable aripiprazole	2	0	(0.0)
Injectionable paliperidone	1	0	(0.0)

^a^ Includes monotherapy or combination therapy of typical antipsychotics only; concomitant use of atypical antipsychotics is not included.

^b^ Includes monotherapy or combination therapy of atypical antipsychotics only; concomitant use of typical antipsychotics is not included.

Among 66 women exposed to any typical antipsychotics, the prevalence of overall MCMs was 4.5% (*n* = 3): 1 of 3 women exposed to oral prochlorperazine (33.3%), 1 of 7 exposed to oral haloperidol (14.3%), and 1 of 16 exposed to oral levomepromazine (6.3%). Among 271 women exposed to any atypical antipsychotics, the prevalence was 8.1% (*n* = 22): 3 of 17 exposed to oral perospirone (17.7%), 6 of 59 to oral quetiapine (10.2%), 5 of 52 to oral risperidone (9.6%), 2 of 23 to oral blonanserin (8.7%), 8 of 123 to oral aripiprazole (6.5%), and 3 of 57 to oral olanzapine (5.3%). Among 46 women exposed to only typical antipsychotics, the prevalence was 4.3% (*n* = 2), and among 251 women exposed to only atypical antipsychotics, it was 8.4% (*n* = 21). No MCMs were observed in women exposed to oral sulpiride, chlorpromazine, bromperidol, fluphenazine, perphenazine, propericiazine, tiapride, paliperidone, asenapine or any injectionable antipsychotics during the first‐trimester. The prevalence of overall MCMs by individual antipsychotic monotherapy is shown in Table S4.

First‐trimester exposure to any antipsychotics was not significantly associated with overall MCM after adjustment using propensity‐score overlap weights (wOR 1.144, 95% CI 0.727–1.799, Table 3). First‐trimester exposure to any atypical antipsychotic was not significantly associated with overall MCM after adjustment using propensity‐score overlap weights (wOR 1.251, 95% CI 0.781–2.007, Table 3). Logistic regression analyses for any typical antipsychotics and for each individual antipsychotic were not conducted because of fewer than 10 outcome events in either the exposed or unexposed groups.

**TABLE 3:**
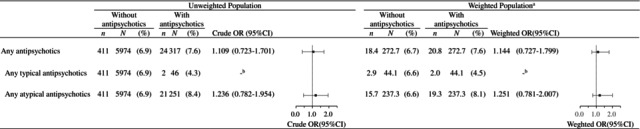
Prevalence and risk of overall major congenital malformations associated with first‐trimester antipsychotic exposure in pregnant women with psychiatric disorders.

Abbreviations: CI, Confidence interval; N, number of women; n, number of overall major congenital malformation; OR, Odds ratio.

^a^ Overlap weights create a balance of mean values of covariates included in the propensity score; therefore, the exposure groups are identical after weighting.

^b^ Analysis was not performed because of fewer than 10 outcome events in either group.

### 3.2 Sensitivity Analyses of the Association Between First‐Trimester Antipsychotic Exposure and Risk of Overall MCMs

Sensitivity analyses were conducted to evaluate the robustness of the main findings (Table 4). These included restricting the analysis to women who were prescribed antipsychotics for ≥ 30 days during the first trimester, varying the exposure window by ±7 days around pregnancy onset, and excluding women with potential teratogenic conditions, including diabetes, obesity, hypertension, and epilepsy, during the first trimester. No significant associations between first‐trimester antipsychotic exposure and risk of overall MCMs were observed in any of these scenarios, and the results were consistent with the main analysis. Furthermore, stratified sensitivity analyses among women diagnosed with schizophrenia, bipolar disorder, or depressive episodes, as well as analyses limited to individual antipsychotic monotherapies, were not conducted using logistic regression because of fewer than 10 outcome events in either the exposed or unexposed groups.

**TABLE 4:**
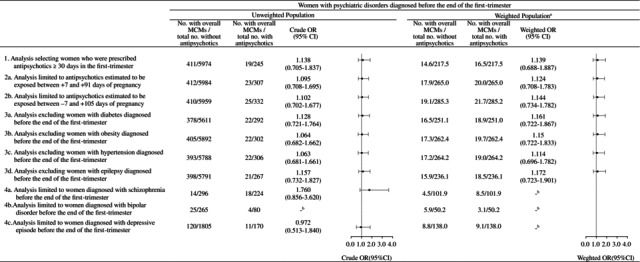
Sensitivity analysis to evaluate the association between the first‐trimester antipsychotic exposure and the risk of overall major congenital malformations.

Abbreviations: CI, Confidence interval; MCMs, Major congenital malformations; OR, Odds ratio.

^a^ Overlap weights create a balance of mean values of covariates included in the propensity score; therefore, the exposure groups are identical after weighting.

^b^ Analysis was not performed due to fewer than 10 outcome events in either group.

## 4 Discussion

### 4.1 Results in Context and Clinical Implications

First‐trimester exposure to any antipsychotics was not significantly associated with overall MCMs in pregnant women with psychiatric disorders (Table 3). This result was consistent across sensitivity analyses, including variations in exposure window, prescription duration, and exclusion of women with comorbidities known to affect fetal development (Table 4). This findings may contribute to discussions on the safety of antipsychotic use during pregnancy, suggesting the importance of carefully balancing the continuation of treatment for maternal psychiatric conditions with potential fetal MCM risk. New insights suggest that neuropsychiatric medications use during pregnancy does not necessarily correlate with behavioral problems in children [51]. Continued antipsychotic use in pregnant women with psychiatric disorders is important to maintain maternal mental health and reduce stress and anxiety.

The mechanisms underlying antipsychotic‐induced teratogenicity remain unclear. However, antipsychotic agents are known to act on neurotransmitter signaling pathways, particularly those mediated by dopamine and serotonin receptors [52]. Neurotransmitters such as dopamine and serotonin are known to play key roles in regulating cell proliferation, differentiation, and apoptosis during embryonic development, particularly during early organogenesis [53].

Reproductive and developmental toxicity studies in animals have shown evidence of congenital malformations following exposure to aripiprazole and to quetiapine [54, 55]. In contrast, no teratogenic effects have been observed with blonanserin, olanzapine, or risperidone in reproductive toxicity studies [56, 57, 58]. Haloperidol has been associated with orofacial clefts in mice and severe cranial malformations in hamsters, and limb malformations have also been reported in human case studies, leading to its contraindication during pregnancy [59]. These findings suggest that antipsychotic drugs could contribute to specific patterns of malformations, even if overall MCM prevalence is not significantly increased. Therefore, our null findings should be interpreted with caution, and further mechanistic studies are warranted to elucidate potential developmental risks. Importantly, most animal studies indicating teratogenic effects have employed doses exceeding human therapeutic levels, and species differences in drug metabolism limit direct extrapolation to human pregnancy. Thus, large‐scale human epidemiological studies remain essential to complement preclinical findings and clarify real‐world teratogenic risks. However, while continuing antipsychotics during pregnancy is essential, the potential risk of fetal congenital malformations should be considered. Although the overall teratogenic risk of antipsychotics is considered low [18, 60, 61, 62], a few studies have reported a slight increase in the prevalence of MCMs [63, 64].

In the present study, the prevalence of MCMs was 6.9% in pregnancies unexposed to any antipsychotics, 7.6% in pregnancies exposed to any antipsychotics, 4.3% in pregnancies exposed to typical antipsychotics, and 8.1% in those exposed to atypical antipsychotics. In a Danish cohort study, the prevalence of MCMs was reported to be 4.9% in pregnancies unexposed to antipsychotics and 7.2% in those exposed to any antipsychotics [61]. In a Hong Kong cohort study, the prevalence of MCMs was 4.9% among pregnancies unexposed to antipsychotics, 6.2% among those exposed to typical antipsychotics, and 9.1% among those exposed to atypical antipsychotics [65]. In another multinational study conducted in Nordic countries and the United States, the corresponding prevalences were 2.7%, 3.1%, and 4.3%, respectively, for pregnancies unexposed to antipsychotics, exposed to typical antipsychotics, and exposed to atypical antipsychotics [18]. These prevalence differences may reflect variations in methodology, population, or sampling periods. For example, the MCM sampling period for live‐born infants differed; while the multinational study conducted in Nordic countries and the United States evaluated congenital malformations diagnosed within the first 3 months of life [18], our study targeted the first year of life. However, the overall safety profile of these medications in pregnancy remains consistent with previous studies, and the benefits of continuing antipsychotic treatment during pregnancy to prevent relapse of psychiatric symptoms often outweigh the potential risks. A recent systematic review evaluated the risks of congenital malformations associated with antipsychotic use during pregnancy [52]. This review suggests that antipsychotic use during pregnancy does not substantially increase the risk of congenital malformations and should not be withheld from patients requiring these medications. However, although this review included populations from various countries, only one study involved Japanese patients, and the antipsychotic types varied by country. Given these differences, the findings of Japanese studies should be further considered. In Japan, although perinatal pharmacoepidemiology studies are becoming increasingly systematized with expanded data sources and methodological frameworks [66], reports on antipsychotic use during pregnancy and its association with congenital malformations are limited [16]. The study indicated that exposure to atypical antipsychotics during the first trimester did not significantly increase the risk of congenital malformations. Nonetheless, its limitations include a relatively small sample size and potential confounding factors, such as the influence of other medications or underlying maternal conditions, which may affect the outcomes [23, 67].

### 4.2 Strengths and Limitations

To minimize confounding by indication, we selected pregnant women with psychiatric disorders, further categorizing them into schizophrenia, bipolar disorder, and depressive episodes, the main indications for antipsychotics. We evaluated the association between antipsychotic exposure and MCMs. To prevent recall bias and to avoid limiting the analysis to patients from particular healthcare institutions, we used a claims database. Pregnant women with psychiatric disorders who were not exposed to antipsychotics served as the control group, but background characteristics may differ between those who require antipsychotics and those who do not. To adjust for these differences, we used overlapping weights for robust epidemiological assessment.

This study had certain limitations. First, it was impossible to confirm whether the prescribed drugs were being used. Second, women with miscarriages or stillbirths were not evaluated, which requires separate consideration. Studies have shown that 40%–70% of spontaneously aborted embryos or fetuses exhibit malformation [68]. Third, medication use during pregnancy may cause miscarriage owing to such malformations. As some recent studies have evaluated the risk of miscarriage, we focused on live‐born infants in this study. A study by Sakai et al. using the Japanese Adverse Event Database reported a potential signal for miscarriage associated with the use of aripiprazole during pregnancy [69]. No miscarriage signals were detected with atypical antipsychotics, and early pregnancy antipsychotic use showed limited or no teratogenic effects [21]. Fourth, the study participants were limited to linked mother–child pairs, potentially limiting the generalizability of the results. Further studies on a generalizable population are required. Finally, this study may have been unable to adequately evaluate the findings because of the insufficient sample size. Although this analysis was conducted, the limited sample size may have compromised the utility of the results. Among pregnant women exposed to individual antipsychotic monotherapies, logistic regression analyses could not be performed because fewer than 10 outcome events were observed in any of the exposed groups (Table 3). Nevertheless, there was no clear trend indicating a markedly higher prevalence of MCMs in most exposure groups compared with their respective unexposed counterparts. Although our primary analysis was restricted to pregnant women with psychiatric disorders to address confounding by indication, residual confounding may still occur between antipsychotic‐exposed and unexposed groups (Table S3). Therefore, additional sensitivity analyses were conducted among women diagnosed with schizophrenia, bipolar disorder, and depressive episodes. These sensitivity analyses yielded similar results. No MCMs were observed among women exposed to certain typical antipsychotics (sulpiride, chlorpromazine, haloperidol, and bromperidol); however, formal statistical comparisons were not feasible because of the limited sample size, restricting drug‐specific risk interpretation. The number of MCMs with and without antipsychotic mothers is negligible, making it difficult to assess the associations between antipsychotics during the first‐trimester and MCMs. We used the largest available cohort; however, future studies with larger sample sizes must ensure more robust and reliable evaluations.

### 4.3 Conclusions

This study did not suggest an obvious association between early pregnancy exposure to antipsychotics and the risk of Overall MCMs in infants born to mothers with psychiatric disorders in Japan.

## Author Contributions

Ryo Obara, Takamasa Sakai, Tomofumi Ishikawa, Kei Morishita, Motohiko Adomi, Azusa Hara, Saya Kikuchi, Natsuko Kobayashi, Noriyuki Iwama, and Taku Obara were responsible for study conception and design and the interpretation of the results. Ryo Obara was responsible for the analysis and drafting the manuscript. Takamasa Sakai and Motohiko Adomi provided statistical advice and contributed to the interpretation of the results. Takamasa Sakai, Tomofumi Ishikawa, Kei Morishita, Motohiko Adomi, Azusa Hara, Saya Kikuchi, Natsuko Kobayashi, Noriyuki Iwama, Genki Shinoda, Aoi Noda, Masatsugu Orui, Mami Ishikuro, Hiroaki Tomita, Nariyasu Mano, Shinichi Kuriyama, and Taku Obara contributed to the clinical interpretation and critical revision of the manuscript. Taku Obara provided advice on essential intellectual content and helped draft the manuscript. All authors read and approved the final version of the manuscript.

## Funding

This work was supported by grants from the Ministry of Health, Labour and Welfare of Japan (H23‐iyaku‐ippan‐006), the Research Institute of Healthcare Data Science, and the Japan Society for the Promotion of Science‐Grant Number (JSPS KAKENHI JP 25K13625). The funders had no role in the study design, data analysis, or publication.

## Ethics Statement

The Institutional Review Board of Tohoku University School of Medicine approved this study on July 19, 2016 (registration number: 2016‐1‐230), waiving the need for informed consent.

## Conflicts of Interest

Tomofumi Ishikawa is an employee of Pfizer R&D Japan and a research collaborator at Tohoku University and has contributed to the present study independently of Pfizer R&D Japan. Nariyasu Mano received presentation fees from Daiichi Sankyo Co. Ltd. The other authors have no conflicts of interest to declare.

## Supporting information


**Table S1:** Antipsychotics investigated in the current study.
**Table S2:** List of covariates considered in this study.
**Table S3:** Characteristics of women with psychiatric disorders with or without antipsychotic exposure.
**Table S4:** Prevalence of major congenital malformations in the study population with antipsychotic monotherapy exposure.

## Data Availability

The data that supports the findings of this study are available in the Supporting Information S1 of this article.
