# Supplementary material for: Risk of Major Congenital Malformations Associated With the First‐Trimester Exposure to Antipsychotics: A Large Claims Database Study
Source: Pharmacol Res Perspect. 2026 Mar 11;14(2):e70231. doi: 10.1002/prp2.70231 (PMC13140567; doi:10.1002/prp2.70231)
Supplement: Supplementary file 1 — Table S1: Antipsychotics investigated in the current study. Table S2: List of covariates considered in this study. Table S3: Characteristics of women with psychiatric disorders with or without antipsychotic exposure. Table S4: Prevalence of major congenital malformations in the study population with antipsychotic monotherapy exposure. [file PRP2-14-e70231-s001.docx]

**SUPPLEMENTARY APPENDIX**

[**Estimation of pregnancy onset and delivery** 2](#_Toc212158733)

[**Sensitivity analyses** 5](#_Toc212158734)

[**Table S1. Antipsychotics investigated in the current study** 7](#_Toc212158735)

[**Table S2. List of covariates considered in this study** 8](#_Toc212158736)

[**Table S3. Characteristics of women with psychiatric disorders with or without antipsychotic exposure** 10](#_Toc212158737)

[**Table S4. Prevalence of major congenital malformations in the study population** **with antipsychotic monotherapy exposure** 11](#_Toc212158738)

# **Estimation of pregnancy onset and delivery**


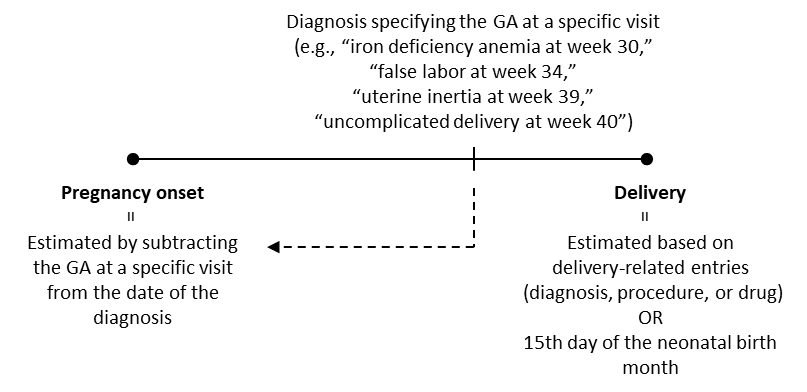


Abbreviations: GA, Gestational age

The date of pregnancy onset was estimated by subtracting the gestational age (GA) recorded as part of the diagnosis at a specific visit from the date of diagnosis. For example, when “delivery at week 39” was entered, 39 weeks and 0 days were subtracted. If a woman made several visits with diagnoses specifying the GA, the longest GA was applied, given that the GA at delivery represents the best obstetric estimate for clinical care ^1,2^; thus, the GA likely has greater accuracy in later stages of pregnancy. Based on an assessment using administrative data from a university hospital, 92.8% of the estimated pregnancy onset dates were within ±7 days of the gold standard date of pregnancy onset ^3^.

The delivery date was estimated based on delivery-related entries described in our previous reports and the birth months of infants ^3–5^. An algorithm was developed wherein the earliest dates of the selected diagnoses and surgical procedures were regarded as the delivery dates. In patients without such diagnoses or procedures, the earliest dates of any other delivery-related entries, including diagnoses, surgical or medical procedures, or injectable medications, were considered delivery dates. Based on an assessment using administrative data from a university hospital, 96.4% of the estimated delivery dates were within ±7 days of the gold standard delivery date ^3^. Given that birth months and years were available, delivery-related entries were used when the date was within the infant’s birth month. Delivery is not always covered by health insurance in Japan, and related information is not recorded when a delivery does not require procedures or medications that are covered by health insurance. In such patients, the 15th day of the neonatal birth month was considered the delivery date.

Labor induction is recommended in post-term pregnancies (pregnancies that reach or extend beyond 294 days of gestation) because of the increased risk of perinatal mortality ^2^. Thus, when the difference between the estimated dates of pregnancy onset and delivery exceeded 294 days, a gestational period of 294 days was uniformly assigned, and pregnancy onset was considered 294 days before the estimated delivery date. Pregnancies were divided into first (pregnancy onset to week 13 day 6 of gestation), second (week 14 day 0 to week 27 day 6), and third (week 28 day 0 to delivery) trimesters ^2^.

Reference:

1. Committee on Obstetric Practice, the American Institute of Ultrasound in Medicine, Society for Maternal-Fetal Medicine. Committee Opinion No 700: Methods for estimating the due date. *Obstet Gynecol.* 2017;129(5):e150-e154. <https://doi.org/10.1097/aog.0000000000002046>
2. Japan Society of Obstetrics and Gynecology and Japan Association of Obstetricians and Gynecologists. Guidelines for obstetrical practice in Japan 2020 edition. Tokyo: Japan Society of Obstetrics and Gynecology; 2020 (in Japanese).
3. Ishikawa T, Obara T, Nishigori H et al. Development of algorithms to determine the onset of pregnancy and delivery date using health care administrative data in a university hospital in Japan. *Pharmacoepidemiol Drug Saf.* 2018;27(7):751-762. <https://doi.org/10.1002/pds.4444>
4. Ishikawa T, Obara T, Nishigori H et al. Antihypertensives prescribed for pregnant women in Japan: Prevalence and timing determined from a database of health insurance claims. *Pharmacoepidemiol Drug Saf.* 2018;27(12):1325-1334. <https://doi.org/10.1002/pds.4654>
5. Ishikawa T, Obara T, Jin K et al. Examination of the prescription of antiepileptic drugs to prenatal and postpartum women in Japan from a health administrative database. *Pharmacoepidemiol Drug Saf.* 2019;28(6):804-811. <https://doi.org/10.1002/pds.4749>

# **Sensitivity analyses**

Several sensitivity analyses of the overall major congenital malformations (MCMs) were conducted in the study population. First, the analyses were repeated by selecting only women prescribed antipsychotic medications for ≥ 30 days in the first-trimester. Second, antipsychotic exposure was estimated to be between (a) +7 and +91, and (b) –7 and +105 days of pregnancy onset was considered instead of first‑trimester exposure, given that the onset of the pregnancy was estimated with an accuracy of ±7 days ^1^. Third, analyses excluding women with diabetes, obesity and hypertension before the end of the first-trimester were conducted, as these disease has teratogenic effects and significantly increases the risk of major congenital disabilities ^2,3^. Finally, analyses excluding women with epilepsy before the end of the first-trimester were performed, as epilepsy is considered a maternal condition that can interfere with normal child development ^2^.

We restricted the main analysis to women diagnosed with any psychiatric disorder (ICD‑10 F00–F99), which includes schizophrenia (F20), bipolar disorder (F31), and depressive episode (F32), to minimize confounding by indication. Additionally, given the disproportionate representation of schizophrenia, bipolar disorder, and depressive episode diagnoses in the antipsychotic-exposed group (as shown in Table S4), conducting supplementary stratified sensitivity analyses by primary diagnosis (i.e., schizophrenia, bipolar disorder, depressive episode) may be warranted. This would help assess whether the association between antipsychotic exposure and MCMs differs among diagnostic subtypes, further addressing residual confounding by indication.

To avoid the influence of combination therapy, we also evaluated the risk of MCMs among women exposed to individual antipsychotic monotherapy.

Reference:

1. Ishikawa T, Obara T, Nishigori H et al. Development of algorithms to determine the onset of pregnancy and delivery date using health care administrative data in a university hospital in Japan. *Pharmacoepidemiol Drug Saf.* 2018;27(7):751-762. <https://doi.org/10.1002/pds.4444>

2. Gilbert-Barness E. Teratogenic causes of malformations. *Ann Clin Lab Sci.* 2010;40(2):99-114.

3. Oliveira CI, Fett-Conte AC. Birth defects: Risk factors and consequences. *J Pediatr Genet.* 2013;2(2):85-90. doi: [10.3233/PGE-13052](https://doi.org/10.3233/pge-13052)

# **Table S1. Antipsychotics investigated in the current study**

| Drug class | Drug subclass | Nonproprietary name | WHO-ATC codes^a^ |
| --- | --- | --- | --- |
| Typical antipsychotic | Benzamide | Nemonapride | N05AX^b^ |
|  |  | Sulpiride | N05AL01 |
|  |  | Sultopride | N05AL02 |
|  |  | Tiapride | N05AL03 |
|  | Butyrophenone | Bromperidol | N05AD06 |
|  |  | Haloperidol | N05AD01 |
|  |  | Pimozide | N05AG02 |
|  |  | Pipamperone | N05AD05 |
|  |  | Spiperone | N05AX^b^ |
|  |  | Timiperone | N05AX^b^ |
|  | Iminodibenzyl | Clocapramin | N05AX^b^ |
|  |  | Mosapramine | N05AX10 |
|  | Indole | Oxypertin | N05AE01 |
|  | Phenothiazine | Carpipramine | N05AX^b^ |
|  |  | Chlorpromazine | N05AA01 |
|  |  | Fluphenazine | N05AB02 |
|  |  | Levomepromazine | N05AA02 |
|  |  | Perphenazine | N05AB03 |
|  |  | Prochlorperazine  (Excluding injectable drugs) | N05AB04 |
|  |  | Propericiazine | N05AC01 |
|  |  | Thioridazine | N05AC02 |
|  | Serotonin-dopamine antagonist | Zotepine | N05AX11 |
| Atypical antipsychotic | Dopamine partial agonist | Aripiprazole | N05AX12 |
|  |  | Brexpiprazole | N05AX16 |
|  | Multi-acting receptor-targeted antipsychotic | Asenapine | N05AH05 |
|  |  | Clozapine | N05AH02 |
|  |  | Olanzapine | N05AH03 |
|  |  | Quetiapine | N05AH04 |
|  | Serotonin-dopamine antagonist | Blonanserin | N05AX^b^ |
|  |  | Lurasidone | N05AE05 |
|  |  | Paliperidone | N05AX13 |
|  |  | Perospirone | N05AX^b^ |
|  |  | Risperidone | N05AX08 |

Abbreviations: ATC = Anatomical Therapeutic Chemical, WHO = World Health Organization

^a^In this study, antipsychotics were identified by their nonproprietary names; however, the corresponding ATC codes are provided in this table.
^b^A specific code is unavailable for these drugs.

# **Table S2. List of covariates considered in this study**

| **Covariates** | **Definition** | **Rationale** | |
| --- | --- | --- | --- |
| **Maternal characteristics and pregnancy outcomes** | | |  |
| Maternal age at delivery | Maternal age at delivery | Maternal age is a known risk factor for BDs.^1,2^ | |
| Delivery year | Calendar year of delivery | This factor partly addresses residual confounding, given the long follow-up period of the JDMC claims database. | |
| **Maternal comorbidities^a^** | | |  |
| Diabetes | ICD-10 code E10–E14^3^, O24 | Diabetes has teratogenic effects and significantly increases the risk for major BDs.^1,2^ | |
| Hypertension | ICD10 code O10-O16, I10-I15 | During pregnancy, hypertension is associated with an increased risk of BDs.^1^ | |
| Obesity | ICD-10 code E66^3^ | During pregnancy, obesity is associated with an increased risk of BDs.^1^ | |
| Epilepsy | ICD-10 code G40, G41^4^ | Epilepsy is considered a maternal disease that can interfere with originally normal child development.^1^ | |
| Phenylketonuria | ICD-10 code E700, E701^5^ | Maternal phenylketonuria leads to defects.^1^ | |
| **Diseases and conditions^b^** |  | | |
| Certain infectious and parasitic diseases | A00-B99 | Maternal diseases and conditions could potentially affect maternal health and pregnancy outcomes, excluding overall MCMs in infants. | |
| Neoplasms | C00-D48 |  |  |
| Diseases of the blood and blood-forming organs | D50-D89 |  |  |
| Endocrine, nutritional, and metabolic diseases | E00-E90 |  |  |
| Diseases of the nervous system | G00-G99 |  |  |
| Diseases of the eye and adnexa | H00-H59 |  |  |
| Diseases of the ear and mastoid process | H60-H95 |  |  |
| Diseases of the circulatory system | I00-I99 |  |  |
| Diseases of the respiratory system | J00-J99 |  |  |
| Diseases of the digestive system | K00-K93 |  |  |
| Diseases of the skin and subcutaneous tissue | L00-L99 |  |  |
| Diseases of the musculoskeletal system and connective tissue | M00-M99 |  |  |
| Diseases of the genitourinary system | N00-N99 |  |  |
| Pregnancy, childbirth, and the puerperium | O00-O99 |  |  |
| Certain conditions originating in the perinatal period | P00-P96 |  |  |
| Congenital malformations, deformations, and chromosomal abnormalities | Q00-Q99 |  |  |
| Symptoms, signs, and abnormal clinical and laboratory findings not elsewhere classified | R00-R99 |  |  |
| Injury, poisoning, and certain other consequences of external causes | S00-T98 |  |  |
| Codes for special purposes | U00-U99 |  |  |
| External causes of morbidity and mortality | V01-Y98 |  |  |
| Factors influencing health status and contact with health services | Z00-Z99 |  |  |

| **Concomitant medication^a^** | | |  |
| --- | --- | --- | --- |
| **Factors associated with disease burden of all disease** | | |  |
| No. of prescribed medications in the first trimester | The number of distinct drugs prescribed in the first trimester, with counting based on generic names | General markers of illness burden^6^ | |
| No. of diagnoses before the end of the first trimester | The number of distinct diagnoses based on ICD-10 block (e.g., A00–A09) | General markers of illness burden^6^ | |
| Abbreviations: BD = birth defect, CA = congenital abnormalities, ICD-10 = International Classification of Diseases, MCM = major congenital malformation  **^a^**Maternal comorbidities and medications listed below that are considered teratogenic causes^1,2^ were not considered covariates in this study for the following reasons.   - Hyperthermia (defined as a body temperature of ≥38.9 °C)/hypothermia (defined as a core body temperature of <35 °C): not evaluable due to the lack of body temperature data. - Alcohol-related disorders: the number of women with the diagnoses (ICD-10 code F10) is minimal; thus, the diagnoses do not seem to reflect alcohol consumption status appropriately in the Japanese claims data. - Folic acid drugs prescribed in the first trimester: folic acid supplements could not be captured in the claims database.   **^b^**Diseases and conditions   - ICD-10 chapters as covariates, excluding F00-F99 (mental and behavioral disorders).   References:   1. Gilbert-Barness E. Teratogenic causes of malformations. *Ann Clin Lab Sci.* 2010;40(2):99-114. 2. Oliveira CI, Fett-Conte AC. Birth defects: Risk factors and consequences. *J Pediatr Genet*. 2013;2(2):85-90. doi: [10.3233/PGE-13052](https://doi.org/10.3233/pge-13052) 3. Quan H, Sundararajan V, Halfon P, et al. Coding algorithms for defining comorbidities in ICD-9-CM and ICD-10 administrative data. *Med Care.* 2005;43(11):1130-1139. doi: [10.1097/01.mlr.0000182534.19832.83](https://doi.org/10.1097/01.mlr.0000182534.19832.83) 4. Mbizvo GK, Bennett KH, Schnier C, Simpson CR, Duncan SE, Chin RFM. The accuracy of using administrative healthcare data to identify epilepsy cases: A systematic review of validation studies. *Epilepsia.* 2020;61(7):1319-1335. doi: [10.1111/epi.16547](https://doi.org/10.1111/epi.16547) 5. Trefz KF, Muntau AC, Kohlscheen KM, et al. Clinical burden of illness in patients with phenylketonuria (PKU) and associated comorbidities - a retrospective study of German health insurance claims data. *Orphanet J Rare Dis.* 2019;14(1):181. doi: [10.1186/s13023-019-1153-y](https://doi.org/10.1186/s13023-019-1153-y) 6. Huybrechts KF, Bateman BT, Hernández-Díaz S. Use of real-world evidence from healthcare utilization data to evaluate drug safety during pregnancy. *Pharmacoepidemiol Drug Saf.* 2019;28(7):906-922. doi: [10.1002/pds.4789](https://doi.org/10.1002/pds.4789) | | |  |

# **Table S3. Characteristics of women with psychiatric disorders with or without antipsychotic exposure**

**(the study population;** **non-adjusted variables and information not used for covariate adjustment)^†^**

|  | Women with psychiatric disorders in the first trimester | | | | | |
| --- | --- | --- | --- | --- | --- | --- |
|  | Unweighted Population | | | | | |
|  | Without antipsychotics  in the first trimester | |  | With antipsychotics  in the first trimester | | |
|  | (N= | 5974) |  | (N= | | 317) |
|  | n | (%) |  | n | | (%) |
| Gestational age at delivery (days), mean (SD) | 269.3 | (3.0) |  | 270.0 | | (12.8) |
| Preterm birth(<37 weeks) | 783 | (13.1) |  | 43 | | (13.6) |
| Cesarean section | 1501 | (25.1) |  | 95 | | (30.0) |
| Newborn sex |  |  |  |  | |  |
| Male | 3042 | (50.9) |  | 180 | | (56.8) |
| Female | 2932 | (49.1) |  | 137 | | (43.2) |
| Psychiatric disorders Indicated for antipsychotics |  |  |  | |  | |
| Schizophrenia (F20) | 296 | (5.0) |  | 224 | | (70.7) |
| Bipolar disorder (F31) | 265 | (4.4) |  | 80 | | (25.2) |
| Depressive episode (F32) | 1805 | (30.2) |  | 170 | | (53.6) |

Abbreviations: SD: Standard deviation

**^†^**This table includes variables not used for covariate adjustment in the main analysis and provides additional information on pregnancy outcomes beyond MCMs.

# **Table S4. Prevalence of major congenital malformations in the study population** **with antipsychotic monotherapy exposure**

| Women with psychiatric disorders before the end of the first-trimester | |  | Total No.^†^ | No. of Events | (%) |
| --- | --- | --- | --- | --- | --- |
|  | Oral antipsychotics |  |  |  |  |
|  | Oral typical antipsychotics |  |  |  |  |
|  | Oral sulpiride |  | 23 | 0 | (0.0) |
|  | Oral chlorpromazine |  | 6 | 0 | (0.0) |
|  | Oral levomepromazine |  | 4 | 1 | (25.0) |
|  | Oral prochlorperazine |  | 3 | 1 | (33.3) |
|  | Oral haloperidol |  | 2 | 0 | (0.0) |
|  | Oral fluphenazine |  | 2 | 0 | (0.0) |
|  | Oral perphenazine |  | 2 | 0 | (0.0) |
|  | Oral bromperidol |  | 1 | 0 | (0.0) |
|  | Oral propericiazine |  | 0 | 0 | (0.0) |
|  | Oral tiapride |  | 0 | 0 | (0.0) |
|  | Oral atypical antipsychotics |  |  |  |  |
|  | Oral aripiprazole |  | 90 | 7 | (7.8) |
|  | Oral olanzapine |  | 34 | 1 | (2.9) |
|  | Oral quetiapine |  | 31 | 3 | (9.7) |
|  | Oral risperidone |  | 23 | 2 | (8.7) |
|  | Oral blonanserin |  | 11 | 1 | (9.1) |
|  | Oral perospirone |  | 11 | 0 | (0.0) |
|  | Oral paliperidone |  | 2 | 0 | (0.0) |
|  | Oral asenapine |  | 0 | 0 | (0.0) |
|  | Injectionable antipsychotics |  |  |  |  |
|  | Injectionable typical antipsychotics |  |  |  |  |
|  | Injectionable haroperidol |  | 1 | 0 | (0.0) |
|  | Injectionable atypical antipsychotics |  |  |  |  |
|  | Injectionable paliperidone |  | 1 | 0 | (0.0) |
|  | Injectionable aripiprazole |  | 0 | 0 | (0.0) |

^†^The individual antipsychotics listed represent monotherapy exposures during the first-trimester.
